# Low-grade oncocytic tumor of the kidney—a clinical, pathological, and next generation sequencing-based study of 20 tumors

**DOI:** 10.3389/pore.2025.1612150

**Published:** 2025-06-02

**Authors:** Alex Jenei, Boglárka Pósfai, Borbála Dénes, Áron Somorácz, Gertrud Forika, Attila Fintha, Zsófia Mészáros, Noémi Kránitz, Tamás Micsik, Kornélia Veronika Eizler, Nándor Giba, Dávid Semjén, Dóra Kelemen, Ferenc Salamon, Anna Schubert, Gábor Cserni, Adrienn Hajdu, Luca Varga, Balázs Árvai, Dániel Sztankovics, Anna Sebestyén, Fanni Sánta, Andrea Simon, Helga Engi, Zsombor Melegh, Levente Kuthi

**Affiliations:** ^1^ Department of Pathology and Experimental Cancer Research, Faculty of Medicine, Semmelweis University, Budapest, Hungary; ^2^ Department of Pathology, Albert Szent-Györgyi Medical School, University of Szeged, Szeged, Hungary; ^3^ Eurofins-MedServ Ltd., Budapest, Hungary; ^4^ Pathology Unit, Győr-Moson-Sopron Shire Aladár Petz University Teaching Hospital, Győr, Hungary; ^5^ Pathology Unit, Fejér Shire Saint George University Teaching Hospital, Székesfehérvár, Hungary; ^6^ Department of Pathology, Medical School and Clinical Centre, University of Pécs, Pécs, Hungary; ^7^ Pathology Unit, Uzsoki Street Hospital, Budapest, Hungary; ^8^ Pathology Unit, Bács-Kiskun Shire Teaching Hospital, Kecskemét, Hungary; ^9^ Medipredict Ltd., Budapest, Hungary; ^10^ Department of Radiology, Brigham and Women’s Hospital, Harvard Medical School, Boston, MA, United States; ^11^ Department of Surgical and Molecular Pathology, Tumor Pathology Center, National Institute of Oncology, Budapest, Hungary; ^12^ HUN-REN-ONKOL-TTK-HCEMM Oncogenomics Research Group, National Institute of Oncology, Budapest, Hungary

**Keywords:** kidney tumor, LOT, WES, NGS, mTOR pathway

## Abstract

Low-grade oncocytic tumor (LOT) of the kidney is a recently recognized renal neoplasm with distinctive morphologic, immunophenotypic, and molecular features that distinguish it from other eosinophilic tumors such as oncocytoma and chromophobe renal cell carcinoma (chRCC). This study presents a comprehensive analysis of 20 LOTs from 19 patients, integrating clinicopathological, immunohistochemical, and genetic data. LOTs typically appeared as small, unilateral, well-circumscribed tumors with a tan-brown cut surface, composed of uniform eosinophilic cells with round nuclei and occasional perinuclear halos. Key histological hallmarks included an extensive capillary network and central edematous areas without necrosis or significant atypia. Immunohistochemically, all tumors showed strong diffuse CK7 positivity and CD117 negativity, with universal expression of GATA3, GPNMB, and L1CAM. Whole-exome and panel-based sequencing revealed recurrent mutations in the mTOR signaling pathway, including *MTOR*, *TSC1*, and *ATM* genes. mTORC1 activation was confirmed immunohistochemically in one case. No evidence of aggressive behavior or metastasis was observed during the follow-up period (median: 4.5 years). Comparative analysis demonstrated that LOT patients were diagnosed at an older age than those with chRCC and had smaller tumors overall. This study reinforces the notion that LOT is a distinct renal tumor entity with consistent morphology, immunoprofile, and mTOR-pathway-related genetic alterations. Despite overlapping features with other eosinophilic renal neoplasms, the specific immunohistochemical profile and indolent clinical course support LOT’s classification as a unique diagnostic category.

## Introduction

Low-grade oncocytic tumor (LOT) of the kidney is a recently discovered emerging entity that further expands the colorful diagnostic palette of the eosinophilic renal neoplasms or so-called “pink tumors” [1, 2]. However, there is increasing data supporting the distinct nature of these tumors, and some authors have already raised questions regarding the legitimacy of recognizing LOT as a separate subtype [3, 4]. LOTs are unencapsulated, well-circumscribed tumors with a pale brown gross appearance with an average diameter of 30 mm [1]. Microscopically, LOTs are composed of monomorphic tumor cells with oncocytic or eosinophilic cytoplasm, round to oval nuclei with frequent perinuclear halo formation and small but prominent (WHO/ISUP grade 1-2) nucleoli [5]. The growth pattern is variable most often solid, tubular, or trabecular. The characteristic immunophenotype distinguishing LOT from the morphologically overlapping oncocytoma (RO), as well as chromophobe renal cell carcinoma (ChRCC), is CD117 negativity with diffuse strong CK7 expression [1]. LOT lacks complete chromosomal losses or gains frequently observed in ChRCC but often demonstrates deletion of 19p13, 19q13, and 1p36 [1]. Alterations in the mammalian target of the rapamycin (mTOR) pathway are common in sporadic cases [6], as well as in the few published cases of LOT in the setting of tuberous sclerosis [7]. According to the published studies, LOT has an indolent behavior without evidence of metastatic potential [8].

To further understand the nature of this rare and conflicting entity, we present the clinicopathological, immunohistochemical, and genetic findings in 20 cases of LOT.

## Materials and methods

### Study cohort and review process

In this study, tumor resections and nephrectomy specimens were analyzed only; consequently, biopsy samples were excluded. In total, 20 LOT cases in 19 patients were collected from the participating institutes. None of these were earlier published or analyzed in detail. Two pathologists (AJ and LK) performed a final review of the cases with a critical evaluation of the morphology, immunohistochemical features, and molecular genetic data. The main clinical characteristics included were symptoms, age, sex, and any underlying renal disorder. Follow-up data were collected from the electronic patient files and general practitioners. The data on multifocality, laterality, surgical technique, and tumor size were obtained from the original histopathological report. The presence of perinuclear halos, cytoplasmic clearing, edematous areas, and delicate capillary network were reviewed.

### Immunohistochemistry

#### Mapping analysis of all tumors

All immunohistochemical stains were performed at the same laboratory (Department of Pathology, Albert Szent-Györgyi Medical School, University of Szeged), applying Leica Bond-Max Automated IHC Staining System (Leica Biosystems, Deer Park, IL, United States). The antibodies that were used in this study are summarized in Table 1. Primary antibodies were visualized using the Bond Polymer Refine Detection kit (Leica Biosystems). In parallel, we stained appropriate positive and negative controls. The reactions were appreciated in a semiquantitative fashion (<1% positivity of tumor cells: - (negative); 1%–25% positivity of tumor cell: +; 26–50% positivity of tumor cells: ++; 51–75% positivity of tumor cells: +++; and 76-100% positivity of tumor cells: ++++) except for FH, SDHB, and mismatch repair proteins (MMR) because these were evaluated as retained or lost.

**TABLE 1:** The characteristics of the immunohistochemical markers applied.

Antibody	Clone	Source	Dilution
*Mapping analysis*			
CA9	Polyclonal	Novus Biologicals	1:2000
CK7	OVTL12/30	BioSB	1:2000
CD10	56C6	Cell Marque	1:50
AMACR	13H4	BioSB	1:100
Vimentin	V9	Novocastra	1:500
CD117	EP10	BioSB	1:100
MelanA	A103	Labvision	1:200
HMB45	hmb-45	Cell Marque	1:200
CK20	Ks20.8	Cell Marque	1:300
GATA3	L50-823	Cell Marque	1:300
L1CAM	UJ127.11	Merck	1:75
GPNMB	E4D7P	Cell Signaling	1:1,000
SDHB	BSB-131	BioSB	1:200
FH	J-13	Santa Cruz	1:2000
MLH1	ES05	Novocastra	1:100
MSH2	79H11	Novocastra	1:200
MSH6	PU29	Novocastra	1:100
PMS2	EP51	BioSB	1:100
*Investigation of the mTOR pathway*			
Phospho-mTOR (Ser2448)	49F9	CellSignaling	1:100
Phospho-S6 Ribosomal Protein (Ser240/244)	D68F8	CellSignaling	1:100
Rictor	A500-002A	Bethyl Laboratories	1:500
Phospho-Akt (Ser473)	D9E	CellSignaling	1:100
PTEN	D4.3	CellSignaling	1:100
Phospho-p70 S6 Kinase (Thr389)	Polyclonal	CellSignaling	1:100
Phospho-4E-BP1 (Thr37/46)	236B4	CellSignaling	1:1,000
Anti-LKB1 antibody (Ley 37D/G6)	sc-32245	Santa Cruz	1:1,000

CA9 indicates carbonic anhydrase 9; CK, cytokeratin 7; CD, cluster of differentiation; AMACR, α-methylacyl-CoA, racemase (P504S); HMB45, human melanoma black 45; GATA3, GATA, binding protein 3; L1CAM, L1 cell adhesion molecule; GPNMB, glycoprotein nonmetastatic B; SDHB, succinate dehydrogenase B; FH, fumarate hydratase; MLH1, MutL protein homolog 1; MSH2, MutS homolog 2; MSH6, MutS homolog6; and PMS2, postmeiotic segregation increased 2.

#### Investigation of the mTOR pathway activity in patient 14

To better understand the activation of the mTOR pathway, we applied eight mTOR pathway-related IHC markers, which are also summarized in Table 1. These IHC reactions were simply classified as positive or negative, regardless of the intensity or extent of the reaction.

### Molecular pathological analysis

#### Whole-exome sequencing

Five LOT cases underwent whole-exome sequencing (WES) analysis as previously described [9]. Briefly, ten serial sections of 10-μm thickness per formalin-fixed, paraffin-embedded sample were taken, and deoxyribonucleic acid (DNA) was extracted. DNA concentration was measured by Quant-iT 1x dsDNA HS Assay kit (Thermo Fisher Scientific) with Fluostar Omega (BMG Labtech) plate reader. For WES library construction, Twist Library Preparation EF Kit 2.0 with Universal Adaptor System and Exome 2.0 Panel (Twist Bioscience) was applied. The fragment size distribution of the precapture and postcapture libraries were determined by capillary electrophoresis on LabChip GX Touch HT Nucleic Acid Analyzer by using X-Mark HT Chip and DNA NGS 3K Assay kit (PerkinElmer). The libraries were quantified by Quant-iT 1x dsDNA HS Assay kit (Thermo Fisher Scientific) with Fluostar Omega (BMG Labtech). In average, more than 24 Gbp raw data was generated per sample. Demultiplexing, adapter trimming, Q30-filtering, and somatic variant calling of the sequenced data was performed on Dragen Bio-IT platform (Illumina). Genomic variants of vcf files were annotated by using the Nirvana Software package. Tumor mutational burden (TMB) was calculated by the number of non-synonymous somatic mutations (single nucleotide variants and small insertions/deletions) per mega-base in coding regions [10].

#### Panel-based sequencing

For *patient 14 and 17* a panel-based sequencing was carried out. Nucleic acid isolation was performed using Maxwell RSC DNA/RNA FFPE Kit on Maxwell RSC Instrument (Promega) according to the manufacturer’s instruction. DNA and RNA concentrations were measured using a Qubit Fluorometer with Qubit dsDNA HS Assay and Qubit RNA HS Assay Kit (Thermo Fisher Scientific). Libraries were prepared using the Ion Chef™ System with Ion 540™ Chips (Thermo Fisher Scientific) according to the manufacturer’s instructions. We applied the Oncomine Comprehensive Assay v3 (Thermo Fisher Scientific), and the sequencing was performed using an Ion S5™ Plus Sequencer (Thermo Fisher Scientific). The data were analyzed using the Ion Reporter™ Software (v. 5.18) (Thermo Fisher Scientific).

### Comparison of the clinicopathological data with chromophobe RCC and oncocytoma

We compared LOT, chromophobe RCC, and oncocytoma patients in terms of gender, age, and tumor size. From our archive, we retrieved these parameters for 153 oncocytoma and 158 chromophobe RCC patients.

## Results

### Clinical characteristics and follow-up

The clinicopathological data are summarized in Table 2. In our cohort, we investigated 8 men and 11 women (male-to-female ratio: 1:1.38). The median age was 67 years (mean: 66.3 years; range: 44–83 years). Also, the tumors were treated with nephrectomy and resection in 10 and 9 of the cases, respectively. Apart from *patient 12* and *19*, the surgery was carried out because of a clinically detected tumor. In *patient 12*, the tumor was incidental finding in an end-stage kidney, while in *patient 19*, LOT developed in a graft kidney, which had no function due to chronic antibody-mediated rejection at the time of the surgery. All tumors were sporadic, and no syndromic association was observed. Three patients died from non-cancer-related causes, and one patient lost to follow-up. In addition, the remaining 15 patients were alive without any evidence of disease during the follow-up, ranging from 0.3 to 19.09 years (median: 4.5 years, mean: 5.82 years).

**TABLE 2:** Clinicopathological features of the tumors investigated.

Patient	Age (y)	Sex	Follow-up time (years)	Outcome	Type of surgery	Side	Tumor size (mm)	Perinuclear halo	Cytoplasmic clearing	Edematous/hemorrhagic areas	Capillary network
1	76	Male	9.6	NCRD	Nephrectomy	Right	37	Rare	No	Yes	Yes
2	81	Male	9.1	NCRD	Nephrectomy	Left	51	Rare	No	Yes	Yes
3	83	Male	2.09	ANED	Nephrectomy	Right	105	Rare	No	Yes	Yes
4	51	Female	19.09	ANED	Nephrectomy	Right	90	Rare	No	Yes	Yes
5	62	Female	2.98	ANED	Nephrectomy	Left	40	Rare	No	Yes	Yes
6	83	Male	3.7	NCRD	Resection	Left	39	Rare	Yes	Yes	Yes
7	75	Male	4.5	ANED	Resection	Right	20	No	No	Yes	Yes
8	75	Female	-	LTF	Nephrectomy	Left	30	No	No	Yes	Yes
9	51	Female	1.2	ANED	Resection	Right	23	Diffuse	Yes	Yes	Yes
10	73	Male	1.1	ANED	Resection	Right	18	No	No	No	Yes
11	67	Male	5.6	ANED	Resection	Left	21	Rare	Yes	Yes	Yes
12	67	Female	5.12	ANED	Nephrectomy	Left	7	No	No	Yes	Yes
13	74	Female	1.5	ANED	Resection	Right	20	Rare	No	Yes	Yes
14	44	Female	1	ANED	Resection	Right	71	Diffuse	Yes	Yes	Yes
15	73	Female	0.7	ANED	Nephrectomy	Left	13	No	No	Yes	Yes
16	66	Female	2.5	ANED	Resection	Right	27	Diffuse	No	Yes	Yes
17	47	Male	0.3	ANED	Resection	Right	70	Rare	No	Yes	Yes
18	63	Female	0.3	ANED	Nephrectomy	Right	50	No	No	Yes	Yes
19A	49	Female	3.4	ANED	Graftectomy	-	7	No	No	Yes	Yes
19B	49	Female	3.4	ANED	Graftectomy	-	17	No	No	Yes	Yes

NCRD indicates not a cancer-related death; ANED, alive and no evidence of disease; and LTF, lost to follow-up.

### Morphological aspects

**FIGURE 1:**
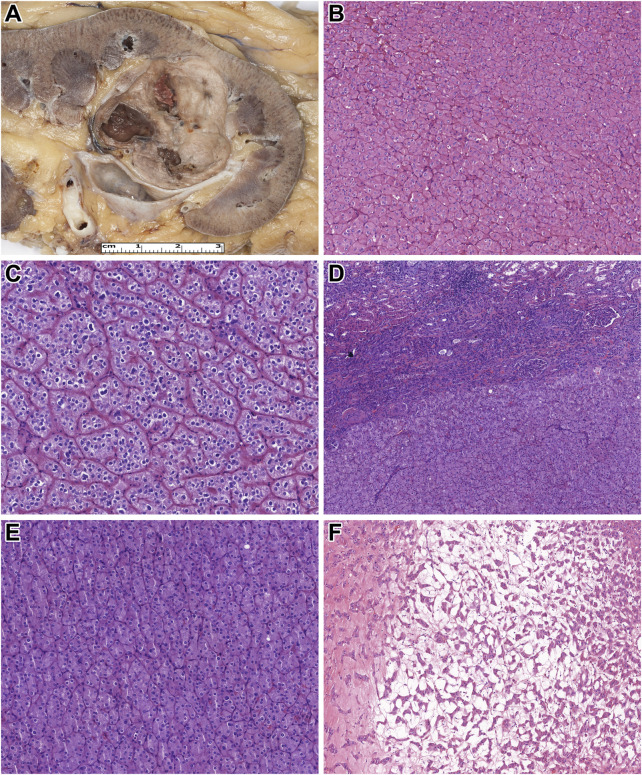
Macroscopic and microscopic features of the tumors studied. **(A)** On the cut surface of the kidney, low-grade onococytic (LOT) tumor is a well-circumscribed, light brown tumor. **(B)** The neoplastic cells are uniform, with eosinophilic cytoplasm and round, centrally located nuclei (H&E, x400). **(C)** A perinuclear halo is observed in some tumor cells (H&E, x400). **(D)** In LOT, typically, no fibrous capsule is present between the tumor tissue and the renal parenchyma (H&E, x200). **(E)** Only a minimal amount of stroma, rich in capillaries, is present among the neoplastic cells (H&E, x400). **(F)** In the central part of LOT, edema and elongated tumor cells are observed (H&E, x200).

**FIGURE 2:**
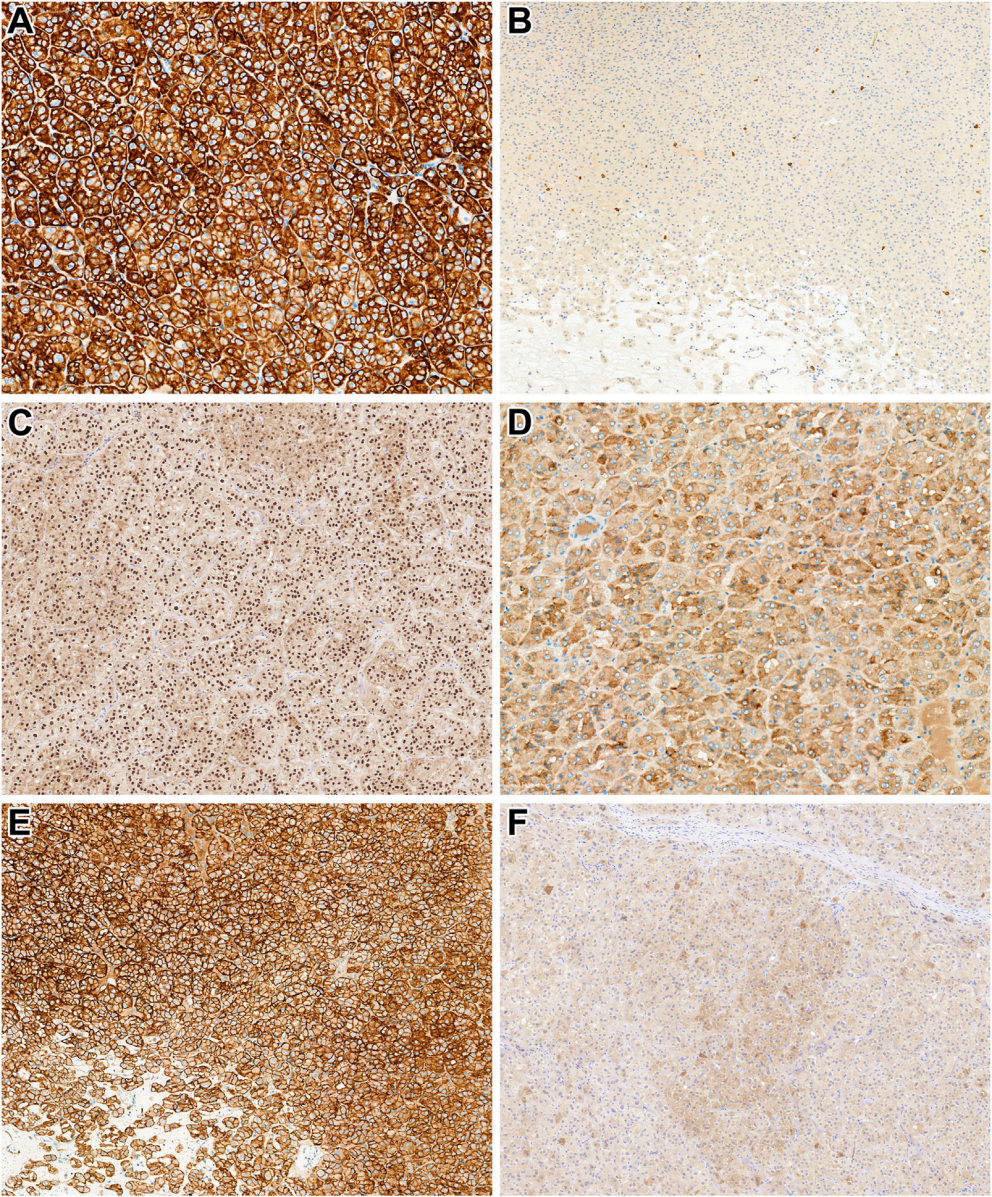
Immunohistochemical characteristics of the tumors investigated. **(A)** In low-grade oncocytic tumor (LOT), CK7 diffusely and strongly (almost in black color) labels the neoplastic cells (x400). **(B)** LOT lacks CD117 expression. Note the mast cells dispersed among the tumor cells, which are positive for CD117 (x200). **(C)** LOT shows diffuse positivity for GATA3 (x200). **(D)** GPNMB is diffusely expressed in the cytoplasm of the tumor cells (x400). **(E)** In LOT, L1CAM is expressed on the membranes of the neoplastic cells (x400). **(F)** Interestingly, MelanA expression was observed in two of our LOT cases (x200).

Here, all tumors studied were unilateral, and all except *patient 19's* tumor were unifocal. The median tumor size was 28.5 mm (mean: 37.8 mm, range: 7–105 mm). Grossly, all tumor formed a solid and well-defined mass with a tan-brown cut surface (Figure 1A). Also, cystic areas, necrosis, and invasion of the extrarenal tissues were not observed. Microscopically, all tumors were built-up by uniform, eosinophilic tumor cells with a round nucleus located in the middle of the cells (Figure 1B). A perinuclear halo was appreciated in 63% of the tumors (Figure 1C), with diffuse presentation in three cases. The predominant growing pattern included solid-nested, trabecular, and rarely tubular. The tumors lacked any pseuocapsule towards the surrounding renal parenchyma (Figure 1D), but towards the fat tissue of the renal sinus and adipose capsule, a thin pseudocapsule was observed. An essential finding was the extensive capillary meshwork among the tumor cells (Figure 1E), that was seen in all tumors. Also, there were well-defined edematous and hemorrhagic areas in all but one tumor, and in these fields, elongated tumor cells formed thin cord-like structures (Figure 1F). It is important to note, that we saw no island-like growing pattern, significant cytological atypia, mitotic figures, and tumor cell necrosis. Regarding the stroma, there was no psammomatous calcification or fibrovascular septa. Immunohistochemically, all cases showed an intense, almost black CK7-positivity and lacked CD117 expression (Figures 2A,B). In addition, GATA3, GPNMB, and L1CAM labelled all tumors (Figures 2C–E), while a variable AMACR expression was seen in 52% of the tumors. A focal MelanA-positivity was appreciated in two tumors (Figure 2F). Also, all tumors were completely negative for CA9, CD10, vimentin, CK20, and HMB45. FH and SDHB were retained in every case. Regarding the MMR proteins, MLH1, MSH2, MSH6, and PMS2 were positive in all tumors, excluding high-level microsatellite instability. The immunohistochemical features are summarized in Table 3.

**TABLE 3:** Immunohistochemical findings of the tumors investigated.

	CA9	CK7	CD10	AMACR	Vimentin	CD117	MelanA	HMB45	CK20	GATA3	L1CAM	GPNMB	SDHB	FH	MMRs
1	-	++++	-	-	-	-	+	-	-	++++	++++	++++	Retained	Retained	Retained
2	-	++++	-	-	-	-	-	-	-	++++	++++	++++	Retained	Retained	Retained
3	-	++++	-	++	-	-	-	-	-	++++	++++	++++	Retained	Retained	Retained
4	-	++++	-	-	-	-	-	-	-	++++	++++	++++	Retained	Retained	Retained
5	-	++++	-	++++	-	-	-	-	-	++++	++++	++++	Retained	Retained	Retained
6	-	++++	-	-	-	-	-	-	-	++++	++++	++++	Retained	Retained	Retained
7	-	++++	-	++++	-	-	-	-	-	++++	++++	++++	Retained	Retained	Retained
8	-	++++	-	++++	-	-	-	-	-	++++	++++	++++	Retained	Retained	Retained
9	-	++++	-	-	-	-	+	-	-	++++	++++	++++	Retained	Retained	Retained
10	-	++++	-	++	-	-	-	-	-	++++	++++	++++	Retained	Retained	Retained
11	-	++++	-	++	-	-	-	-	-	++++	++++	++++	Retained	Retained	Retained
12	-	++++	-	++++	-	-	-	-	-	++++	++++	++++	Retained	Retained	Retained
13	-	++++	-	-	-	-	-	-	-	++++	++++	++++	Retained	Retained	Retained
14	-	++++	-	-	-	-	-	-	-	++++	++++	++++	Retained	Retained	Retained
15	-	++++		-	-	-	-	-	-	++++	++++	++++	Retained	Retained	Retained
16	-	++++	-	+	-	-	-	-	-	++++	++++	++++	Retained	Retained	Retained
17	-	++++	-	++++	-	-	-	-	-	++++	++++	++++	Retained	Retained	Retained
18	ND	++++	ND	ND	ND	-	ND	ND	-	++++	++++	++++	ND	ND	ND
19A	-	++++	-	+	-	-	-	-	-	++++	++++	++++	Retained	Retained	Retained
19B	-	++++	-	-	-	-	-	-	-	++++	++++	++++	Retained	Retained	Retained

CA9 indicates carbonic anhydrase 9; CK, cytokeratin; CD, cluster of differentiation; AMACR, α-methylacyl-CoA, racemase (P504S); HMB45, human melanoma black 45; GATA3, GATA, binding protein 3; L1CAM, L1 cell adhesion molecule; GPNMB, glycoprotein nonmetastatic B; SDHB, succinate dehydrogenase B; FH, fumarate hydratase; and MMR, mismatch repair protein, which includes MLH1, MSH2, MSH6, and PMS2.

### Molecular genetic aspects

All molecular genetic alterations are summarized in Table 4. Whole-exome sequencing was conducted on five patients: *patient 1*, *2*, *3*, *5*, and *9*. For *patient 14* and *17*, panel-based sequencing was performed. In the WES cohort, 1,034 filtered variants were identified. Focusing on genes relevant to tumorigenesis, we detected two *MTOR* mutations, two *ATM* mutations, one *NF1* mutation and one *TSC1* mutation, which are considered pathogenic or likely pathogenic. Notably, in *patient 3*, we detected solely a variant of unknown significance (VUS) in the *MTOR* gene. The TMB was low (<10 mutations per megabase) in all investigated tumors. In the panel-based sequencing cohort, we observed pathogenic mutations in *MTOR* and *ATM* genes.

**TABLE 4:** Genetic findings of the tumors investigated.

Patient	Gene symbol	Variant annotation (p.)	Variant annotation (c.DNA)	Variant allele frequency	Pathogenic role	Molecular consequences	TMB
1	*MTOR* *NF1*	L2427Q P1084L	c.7280T>A c. 3251C>T	11.11% 13.2%	Pathogenic Likely pathogenic	Missense Missense	Low
2	*TSC1* *NF1* *ATM*	I588M I377T Q1503X	c.1764T>G c.1130T>C c.4507C>T	15.38% 30% 11.53%	VUS VUS Pathogenic	- - Nonsense	Low
3	*MTOR*	A138F	c.322_323delinsTT	38%	VUS	-	Low
5	*MTOR* *ATM*	I2500F S1056X	c.7498A>T c.3167C>A	19.71% 18.18%	Likely pathogenic Pathogenic	Missense Nonsense	Low
9	*TSC1*	S682_splice	c.2041 + 1G>C	40.3%	Likely pathogenic	Splice site	Low
14	*ATM*	E2366X*	c.7096G>T	49.55%	Pathogenic	Nonsense	-
17	*MTOR*	L2427Q	c.7280T>A	33%	Pathogenic	Missense	-

TMB indicates tumor mutational burden; and VUS, variant of unknown significance.

### Expression of the mTOR pathway activity proteins in *patient 14*

**FIGURE 3:**
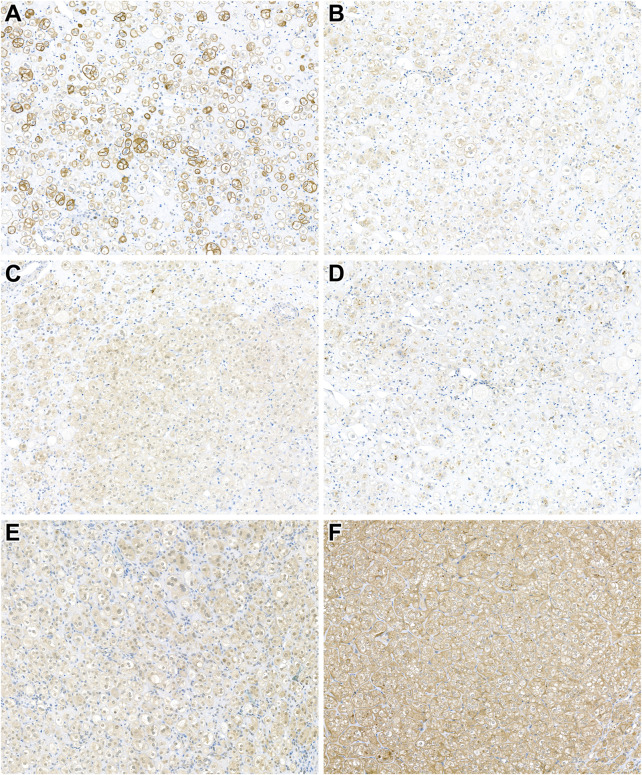
Expression of mTOR pathway-related immunohistochemical markers in *patient 14's* tumor. **(A)** Cytoplasmic phospho-MTOR expression confirms MTOR pathway activation (x400). **(B,C)** Positivity for phospho-4EBP1 and phospho-S6 indicates mTORC1 activity (x400). **(D)** Weak RICTOR expression suggests minimal mTORC2 activity (x400). **(E,F)** PTEN and LKB1 expression is retained in the neoplastic cells (x400).

Figure 3 shows representative images of the reactions carried out. The presence of phospho-MTOR confirms mTOR pathway activation. However, specific markers indicate varying results due to potential technical sensitivities (e.g., phospho-p70S6K negativity). Phospho-4EBP1 and phospho-S6 suggest mTORC1 activity, while minimal mTORC2 activity is observed (weak RICTOR positivity, but negative p-serin-Akt). The LKB1 expression is retained, indicating mTOR activation occurs through a pathway independent of STK11. PTEN positivity suggests no loss of this mTOR regulator in the tumor.

### Correlation with different renal neoplasms

We compared the gender, age, tumor side, and tumor size of LOT patients with those of patients operated on for oncocytoma and chromophobe RCC. No significant difference was observed in gender distribution (*p* = 0.61) or tumor laterality (*p* = 0.79) between the groups. Regarding age, LOT was diagnosed approximately 7 years later than chromophobe RCC [LOT (mean age = 66.3) vs. chRCC (mean age = 59.1), *p* = 0.02]. Oncocytoma appeared roughly 3 years earlier than LOT; however, this difference was not statistically significant [LOT vs. RO (mean age = 63.2), *p* = 0.51]. There was no significant difference in tumor size between LOT and oncocytoma (*p* = 0.57). However, both tumor types were significantly smaller than chromophobe RCC [LOT (mean size = 37.8 mm) vs. chRCC (mean size = 67.7 mm), *p* = 0.001; RO (mean size = 45.1 mm) vs. chRCC, *p* = 0.007].

## Discussion

Chromophobe RCC and oncocytoma are traditionally considered classic eosinophilic renal neoplasms [11]. However, advances in immunoprofiling and genetic analysis have led to the identification of novel and emerging RCC subtypes, including eosinophilic, solid and cystic RCC, eosinophilic vacuolated tumor, etc [2]. In 2019, Trpkov et al. described a distinct subset of 28 renal tumors characterized by unique microscopic features, a specific immunophenotype, and indolent clinical behavior, designating them as low-grade oncocytic tumors [1]. Subsequent studies confirmed that LOT is defined by genetic alterations affecting the mTOR pathway [6, 7]. The primary differential diagnoses for LOT include chromophobe RCC and oncocytoma, though other eosinophilic RCCs, such as succinate dehydrogenase (SDH)-deficient RCC, may also be considered [12]. LOT accounts for less than 1% of all renal cell neoplasms. In a study by Kravtsov et al., LOTs constituted approximately 0.35% of all renal tumors [13]. However, within the subset of eosinophilic renal neoplasms, the incidence of LOT is notably higher, reaching around 5% [14].

Histologically, LOT exhibits a solid peripheral growth pattern with a centrally located edematous area [1]. In most cases, these central regions contain elongated tumor cells, often described as resembling “floating boats on the sea” [7]. In contrast, oncocytoma features fibrotic stroma with an island-like (archipelago-like) growth pattern [15]. Chromophobe RCC, on the other hand, is typically characterized by prominent fibrovascular septa rather than an extensive capillary network [15]. Notably, in our cohort, all LOT cases exhibited a prominent capillary network, and all but one demonstrated central edematous areas.

Cytologically, LOT is composed of uniform tumor cells with small nuclei and eosinophilic cytoplasm [1]. Perinuclear halos, previously described in the literature [7], were observed in our study in 12 tumors, with a rare and diffuse distribution in 75% and 25% of cases, respectively. While extensive perinuclear halos may suggest eosinophilic variant of chromophobe RCC, the characteristic raisinoid nuclei seen in chromophobe RCC were absent in LOT. Furthermore, we did not observe severe cytological atypia or binucleation in our LOT cases, distinguishing them from high-grade eosinophilic renal neoplasms. The absence of these nuclear features also differentiates LOT from oncocytoma, which frequently exhibits some degree of nuclear atypia [1].

Interestingly, in four cases, perinuclear halos were so prominent that they led to cytoplasmic clearing, mimicking the appearance of clear cell RCC. In some instances, this resemblance was striking, further emphasizing the necessity of careful morphological and immunohistochemical assessment in the diagnostic workup of LOT.

According to the literature, LOT exhibits a unique immunophenotypic profile, characterized by CD117 negativity and strong, diffusely positive CK7 expression [1, 2]. This immunostaining pattern effectively distinguishes LOT from oncocytoma and chromophobe RCC. Oncocytoma is diffusely positive for CD117 but typically shows only focal CK7 positivity, often limited to isolated tumor cells [16]. Notably, CK7 expression is more extensive around the central scar in oncocytoma [17]. Chromophobe RCC, by contrast, is diffusely positive for both CD117 and CK7, although in the eosinophilic variant, CK7 staining can be focal [16].

The differential expression of CD117 suggests distinct cellular origins for these tumors. CD117 positivity in oncocytoma and chromophobe RCC indicates their derivation from the intercalated cells of the collecting ducts [18]. The cellular origin of LOT was initially unclear; however, Alghamdi et al. recently demonstrated that L1 cell adhesion molecule (L1CAM) is diffusely expressed in LOT, providing insight into its histogenetic background and serving as a useful diagnostic marker [19]. GATA3 expression, with variable distribution, has also been reported in LOT [7, 20]. While chromophobe RCC may also express GATA3 [21], our findings indicate that both L1CAM and GATA3 label all LOT cases diffusely, suggesting their high sensitivity for this entity [7, 18]. However, the specificity of these markers requires further investigation in larger cohorts.

Interestingly, we identified two LOT cases with MelanA expression, an unusual finding in these neoplasms. Typically, MelanA is expressed in angiomyolipoma (AML), *TFE3*-rearranged RCC (TFE3-RCC), and *TFEB*-altered RCC (TFEB-RCC) [22, 23]. AML, a perivascular epithelioid cell tumor, may enter the differential diagnosis of eosinophilic renal tumors when it has a pure epithelioid morphology, though it lacks cytokeratin expression [22]. TFE3-RCC and TFEB-RCC, classified as molecularly defined renal carcinomas in the current WHO classification, may also express HMB45 but are negative for CK7 [23, 24]. Our findings suggest that aberrant MelanA expression can occasionally be present in LOT.

Additionally, recent studies by Salles et al. identified glycoprotein nonmetastatic B (GPNMB) as a diffusely expressed marker in mTOR-driven renal tumors, making it a promising diagnostic tool [25]. Our analysis confirmed diffuse GPNMB expression in all LOT cases, indicating active mTOR signaling. However, GPNMB is also expressed in TFE3-RCC and TFEB-RCC, limiting its specificity as an entity-defining marker [25]. Rather, its expression reflects underlying genetic alterations in these tumors.

Among eosinophilic renal tumors, SDH-RCC is a low-grade neoplasm that shares some histologic features with LOT [12]. However, key distinguishing characteristics -such as cytoplasmic vacuolization, a tubular growth pattern, and entrapped renal tubules- aid in differentiation at the light microscopic level [26]. Immunohistochemically, SDH-RCC is typically negative for CK7, CD117, and SDHB, further supporting its distinction from LOT [2, 26].

As previously noted, LOTs harbor mutations in the TSC/mTOR pathway, with *MTOR* and *RHEB* being the most frequently altered genes [7]. Mutations in *TSC1*, *TSC2*, *NF2*, and *PIK3CA* have also been reported [8]. However, it remains debated which genetic alterations alone are sufficient to activate mTOR signaling, and which represent passenger mutations. In our series, *MTOR* was mutated in three tumors, while, interestingly, no *RHEB* alterations were detected. A striking finding was the recurrent presence of *ATM* mutations in both the WES and panel-based sequencing cohorts. *ATM* encodes a serine/threonine kinase primarily responsible for detecting DNA double-strand breaks [27]. It initiates homologous recombination repair (HRR) and triggers cell cycle arrest, with downstream effects on the TSC/mTOR pathway [27]. Specifically, activated ATM stimulates LKB1, which in turn activates AMPK. AMPK subsequently phosphorylates TSC2, enhancing its inhibition of mTORC1 [28]. Therefore, ATM mutations in LOT may result in dysregulated TSC/mTOR signaling. However, immunohistochemical findings in *patient 14* appeared contradictory, as the tumor showed retained LKB1 expression. This discrepancy could be explained in two ways. First, LKB1 expression does not guarantee a fully functional protein; with mutated ATM, phosphorylation of LKB1 may be insufficient to activate AMPK. Second, while a single mutation may not significantly disrupt TSC/mTOR signaling, a combination of mutations may have an additive effect, leading to pathway dysregulation and LOT development. The first hypothesis could be tested by assessing phosphorylated LKB1 levels using IHC or Western blotting. The second is supported by our findings, as *ATM* mutations co-occurred with other alterations in our cohort.

*ATM* mutations have been identified in several cancer types, including ovarian, prostate, and lung carcinomas [29]. They have also been reported in clear cell RCC, where reduced ATM expression correlates with poor prognosis [30, 31]. Loss of ATM function causes HRR deficiency, making tumors reliant on alternative DNA repair mechanisms such as base excision repair, which may offer therapeutic opportunities [32]. However, as LOTs typically follow an indolent clinical course, the utility of such therapeutic strategies in this context remains questionable.

In *patient 3*, we identified a VUS in the *MTOR* gene; a similar alteration was reported by Morini et al [5]. Further studies are required to evaluate the pathogenicity of such variants. All analyzed cases demonstrated low TMB, a finding consistent with those reported by Farcaş et al. in eosinophilic vacuolated tumors [33].

LOT tends to arise in slightly older patients compared to more common renal tumors and exhibits a mild female predominance [1, 8]. In our cohort, the patient group comprised nine females and eight males, with a mean age of 67.5 years. This was significantly higher than the mean age of patients with chromophobe RCC in our collection. Additionally, LOTs were significantly smaller in size compared to chromophobe RCCs. However, while LOTs also tended to occur in older patients and were generally smaller than oncocytomas, these differences did not reach statistical significance. Our findings align with previously published data regarding LOT’s demographic and clinicopathological characteristics.

Furthermore, in our study, no cases of tumor relapse or tumor-specific mortality were observed, reinforcing prior reports of the indolent nature of LOT [8]. Currently, the term “low-grade oncocytic tumor” is purely descriptive, referring only to the tumor’s light microscopic appearance. Given the excellent prognosis reported in published cases, an alternative nomenclature, oncocytic principal cell adenoma of the kidney, has been proposed [19]. This designation aims to better reflect both the cellular origin and the benign clinical course of these tumors.

Before concluding, we acknowledge certain limitations of our study. The whole-exome sequencing method we employed did not assess copy number alterations or non-coding (intronic) regions of the genome. In his seminal work on LOT, Trpkov et al. identified deletions in 19p13.3, 19q13.11, and 1p36.33 [1], while Alghamdi et al. reported the presence of an extra chromosome 7 in 41% of analyzed cases [19]. Copy number variations, including gene deletions or amplifications, may contribute to LOT’s tumorigenesis. Additionally, alterations within non-coding regions could impact RNA splicing, disrupt enhancers, silencers, or promoters, and modify microRNA expression or non-coding RNA binding sites. To further elucidate the role of these molecular mechanisms, future studies employing whole-genome sequencing will be necessary.

In this study, we present the clinical, pathological, and genetic characteristics of 20 cases of LOT cases. The epidemiological and pathological findings in our cohort are consistent with previously published data. Immunohistochemically, all tumors demonstrated diffuse expression of GATA3 and L1CAM, reinforcing the diagnostic utility of these markers. Notably, we identified MelanA expression in two cases, a novel finding in LOT. Additionally, GPNMB was diffusely expressed in all tumors, including one case without any detectable genetic alteration. Furthermore, we identified *ATM* mutations, which have not been previously reported in LOT and may represent an alternative genetic mechanism contributing to mTOR activation. The excellent clinical outcomes observed in our study further support the indolent and likely benign-nature of this tumor entity. Although, in terms of the genetic background there is some overlap with other members of other oncocytic renal tumors, overall the results of the present study support the recognition of LOT as a separate entity.

## Data Availability

The raw data supporting the conclusions of this article will be made available by the authors, without undue reservation.
